# Research in the application of artificial intelligence to lung cancer diagnosis

**DOI:** 10.3389/fmed.2024.1343485

**Published:** 2024-01-30

**Authors:** Wenjuan Liu, Nan Shen, Limin Zhang, Xiaoxi Wang, Bainan Chen, Zhuo Liu, Chao Yang

**Affiliations:** ^1^Department of Respiratory and Critical Care Medicine, The First Affiliated Hospital of Dalian Medical University, Dalian, China; ^2^Department of Nephrology, The First Affiliated Hospital of Dalian Medical University, Dalian, China; ^3^Department of Clinical Laboratory, The First Affiliated Hospital of Dalian Medical University, Dalian, China; ^4^Department of Radiology, The First Affiliated Hospital of Dalian Medical University, Dalian, China

**Keywords:** artificial intelligence, lung cancer, diagnosis, pathomics, imageomics, genomics applications

## Abstract

The morbidity and mortality rates in lung cancer are high worldwide. Early diagnosis and personalized treatment are important to manage this public health issue. In recent years, artificial intelligence (AI) has played increasingly important roles in early screening, auxiliary diagnosis, and prognostic assessment. AI uses algorithms to extract quantitative feature information from high-volume and high-latitude data and learn existing data to predict disease outcomes. In this review, we describe the current uses of AI in lung cancer-focused pathomics, imageomics, and genomics applications.

## 1 Introduction

Cancer has become a leading cause of death in the whole world. The burden of cancer is expected to increase as a result of population ageing and changes in lifestyle. Among these, lung cancer has high morbidity and mortality rate, which causes serious burden in the whole word (1, 2). Early diagnosis and personalized treatment are essential to manage this public health issue. Biopsy followed by pathological diagnosis is the gold standard in diagnosis, but chest computed tomography (CT) facilitates early screening and auxiliary diagnosis. Thus, pathologists and radiologists are confronted with greater workloads, lack of human resources, and increasingly stringent diagnostic standards. In recent years, rapid developments in artificial intelligence (AI) have led to its increasing usage in lung nodule detection, including lung cancer diagnosis and prognosis. Several studies (3–8) have demonstrated the combinations of AI and clinical uses in early detection, identification of pathological types, recognition of relevant genetic mutations, and the evaluation of treatments and prognoses. In this review, we describe the basic background and recent developments of AI in these fields.

## 2 Introduction to AI

The terms artificial intelligence (AI) and machine intelligence were initially used in 1956 to refer to computer “intelligence,” which involves mathematics, computer science, and computational neuroscience. AI simulates, extends, or expands human intelligence, aiding the completion of specific tasks. Research focused on computer-aided detection/diagnosis (CADe/Dx) intensified in the 1990s, and CADe/Dx of pulmonary nodules is becoming increasingly accurate. Machine learning is the core component of AI; it refers to the use of computer algorithms and models to draw inferences, recognize data patterns, and accomplish specific tasks without human intervention. The algorithms include shallow learning, deep learning, and other routines. Although shallow learning aids classification and regression, it requires large training datasets and does not adequately manage complex problems. Deep learning, also known as deep neural network learning, uses multi-level feature learning, involving convolutional neural networks (CNNs), deep belief networks, and self-encoders for deep mining of big data; thus, it can solve complex multi-classification problems. Deep learning based on CNNs accurately detects cancerous tissues and malignant tumors, predicts survival, and finds common mutations.

In recent years, imageomics has come to the forefront of AI research. This approach extracts quantitative features from a large number of medical images that reveal tumor morphology, size, and texture, thereby facilitating tumor diagnosis and treatment using new images (9). The steps include image acquisition, lesion outlining, feature extraction, feature selection, and machine modeling (10). Overall, imageomics constitutes task-driven re-use of existing human-labeled image data via both AI and statistical analyses (11); it has applications in imaging suites and nuclear medicine.

AI-assisted diagnosis has led to the concept of pathomics, which involves the transformation of pathology images to high-fidelity, high-throughput mineable datasets with quantitative textural, morphological, and edge gradient features, as well as biological characteristics. These datasets are used to quantify pathological diagnoses and predict disease prognoses (12). Applications of pathomics include cytology, primary screening, quantitative morphological and histopathological diagnoses, and auxiliary prognostic judgments (13). The findings can be compared and integrated with the results of genomic and histological analyses; such comparisons are regarded as multi-omics crossover studies.

Genomics refers to the exploration of relationships between genes and functions using genome-scale assays (14). AI facilitates the complex data analyses, which are necessary for variant identification and classification, imaging and genetic diagnoses, and genotype–phenotype predictions.

## 3 Applications of AI in lung cancer diagnosis

### 3.1 Early differentiation of lung cancer imaging

Approximately 70% of lung cancer patients have middle-stage and late-stage disease at the time of diagnosis; this delayed diagnosis hinders treatment and reduces survival. Early screening is important to reduce mortality. Lung nodules are early signs of lung cancer; early and accurate detection of benign and malignant nodules facilitates early treatment, improving prognosis and survival. Imaging physicians assess nodule location, size, and differences in density relative to surrounding structures. Such evaluations are subjective; the American Lung Cancer Screening Program found that approximately 8.9% of lung cancer cases were missed on baseline CT (15). Zhang et al. (16) compared AI and manual assessments of the chest CT scans of 60 patients with early-stage lung cancer and 50 patients with benign lung nodules (both diagnoses were determined via pathological analyses). The sensitivity of AI in terms of early-stage lung cancer detection was better than the human sensitivity, indicating that the use of AI improved diagnostic sensitivity for early-stage lung cancer and provided rapid results. Clinical implementation was suggested.

Currently, CADe/CADx schemes are used to reduce the false-positive lung nodule rate. CADe first selects all suspected nodules, classifies them as nodules or non-nodules, and removes the non-nodules with high sensitivity. The CADe system is based on deep learning; the Lung Image Database Consortium is used to analyze nodule morphology, density, and texture. Some variations among studies are apparent, which may reflect the nodules examined. Some nodules, such as ground glass nodules (GGNs) and apposed vascular nodules, are associated with high false-positive rates; screening must be improved to enhance detection. CNNs are widely used to screen pulmonary nodules. For example, Mahmood et al. (17) developed a system based on AlexNet, a novel CNN in terms of layer ordering; it exhibits improved hyperparameters and functions. The image segmentation algorithm was used to process the lung scan sequence to generate a lung area map, and then, the lung image was generated according to the lung area map. The lung region image generated by lung segmentation and the nodule labeling information were used to generate the nodule region image, and the pulmonary nodule segmenter based on convolutional neural network was trained, and then, the lung nodule segmentation was performed on the image to obtain the suspected pulmonary nodule area. Once the suspected pulmonary nodule is located, a 3D convolutional neural network is used. The pulmonary nodules were classified to obtain the location and confidence of the true pulmonary nodules (18). An entire dataset is pre-processed, segmented, normalized, and zero-centered. The accuracy is 98.7%, the sensitivity is 98.6%, and the specificity is 98.9%; overall, performance is excellent. Compared with a two-dimensional CNN, a three-dimensional (3D) CNN more effectively analyses spatial information, but its computational burden is larger and a more complex network is involved. Importantly, the sensitivity is higher. Yang et al. (19) used a squeeze excitation module for feature fusion, to solve the problem posed by the different views of a multi-view model; they constructed a 3D multi-view squeeze excitation CNN. The accuracy and sensitivity in terms of classifying benign and malignant pulmonary nodules were 96.04 and 98.59%, respectively; 94.8% of agreement with pathological diagnoses was higher than the agreement achieved by other methods. The model effectively learned the spatial heterogeneities of pulmonary nodules and solved the multi-view discrepancy problem; it will provide substantial assistance to physicians. Ma et al. (20) developed an improved 3D Mask regional CNN for the detection and classification of pulmonary GGNs using a pre-trained 3D U-Net to extract the lung parenchyma, a 3D target detection network to locate the lesion and determine its malignancy status, and a feature-based weighted clustering algorithm to remove false images. The mean detection accuracy was high, and the “false alarm” rate was low. The feature-based weighted clustering algorithm allowed this deep learning-based framework to automatically detect and classify pulmonary GGNs and to accurately locate and classify nodules; it will be useful for physicians. Liu et al. (21) subjected the images of 262 patients with pulmonary GGNs to physician and AI review. The AI sensitivity and leakage rates were lower, but the misdiagnosis rate was higher compared with the rates of the physician. Further cooperation between AI developers and physicians is needed. Wu et al. (22) used AI to analyze images of 175 patients with pulmonary nodules who attended regular follow-up; they found that nodule growth was influenced by original nodule size, mean CT value, surface signs, and malignancy probability. Clinicians were recommended to consider these factors when choosing follow-up times; such considerations will facilitate early detection of nodule growth and timely initiation of treatment.

### 3.2 Lung cancer diagnosis

AI diagnoses of lung cancer include imaging, pathological, and genetic diagnoses. A large number of images are subjected to multi-level quantitative feature extraction, which aids the diagnosis of benign and malignant tumors while predicting histological type and invasiveness; such feature extraction assists clinical diagnosis (23). Yu et al. (24) used a machine learning tool to image lung cancer CT and pathology slices with a focus on lung nodules and segmentation of cell nuclei, as well as digital pathology results. The findings were useful in lung nodule and nuclear screening. The positive diagnostic accuracy was similar to the accuracy of less senior doctors, and the mean screening time was shortened by 58%; thus, the tool improved efficiency, reduced doctor workloads, and supported clinical diagnoses.

### 3.3 AI and CT recognition

AI analyses of medical images improve imaging efficiency and image processing; they also allow remote consultations by primary hospitals (25). Feng et al. (26) evaluated 90 lung cancer patients using a Mask-CNN for image segmentation and a dual path network for nodule detection. In terms of detecting lung lesions, the dual path network accuracy was 88.74%; the accuracy of CT diagnosis was 88.37%, and the sensitivity and specificity were 82.91 and 87.43%, respectively. Notably, CT image examination based on deep learning combined with serum markers exhibited an accuracy of 97.94%, with a sensitivity of 98.12% and a specificity of 100%. Shi et al. (27) used a semi-supervised deep migration learning framework (known as SDTL) for the diagnosis of benign and malignant lung nodules among 3,038 nodules with pathologically confirmed benign or malignant markers and 14,735 unmarked nodules. They found that SDTL exhibited superior diagnostic performance. Migration learning improved the accuracy by 2%, and semi-supervised learning improved the accuracy by an additional 2.9%. Such effective classifications are expected to have applications in clinical practice. Fang et al. (28) analyzed CT images of 224 hairy nodules and GGNs from 210 patients. AI identified GGNs using quantitative parameters and CT signs. The AI parameters identified the early lung adenocarcinoma subtypes, and the combination of AI data with CT signs led to improved diagnostic efficacy.

### 3.4 AI and pathological diagnosis

Many studies have shown that AI can help pathologists to rapidly determine lung cancer types, identify mutations in specific genes, predict prognoses, and make clinical decisions, thereby significantly improving diagnostic efficiency and reducing workloads (29). In a study of 101 patients who had been clinically diagnosed with lung cancer, Wang et al. (30) used an AI cytopathological diagnostic system and rapid on-site evaluation to assess 94 lung tissue biopsy specimen, 6 pleural effusion specimen, and 1 ascites specimen. The AI cytopathological diagnostic accuracy was lower than the accuracy of pathologists but similar to the accuracy of rapid on-site evaluation; this AI system reduced workloads, improved efficiency, and will be useful in lung cancer diagnosis. Chen et al. (31) evaluated 110 lung adenocarcinoma pleural fluid specimen and 20 non-cancerous pleural fluids (controls). They trained the Yolo V4 model using suspected and confirmed lung cancer cells; they trained the Inception V3 model on various classifications of cells. The trained Yolo V4 model identified and labeled both suspected and confirmed lung adenocarcinoma cells in pleural fluids with a mean accuracy of 20% for all classes; in contrast, the accuracy of the trained Inception V3 model was 98%. The dispersal of cell clumps into single cells improved efficiency and accuracy. Thus, AI detected and classified lung adenocarcinoma cells in pleural fluid; this ability aids lung cancer diagnosis and is a good example of a practical application for deep learning.

### 3.5 AI and genotyping

AI can manage the complex big data produced by genomics analyses; it can also assist geneticists and clinicians in accurately evaluating sequencing results. However, AI applications in this field remain limited, and further optimization is needed. Liu et al. (32) used a KL scatter-based gene selection method to identify high-scatter genes. They used the focal loss function to establish a deep neural network and then used k-fold cross-validation (k = 5) to select the best model. The area under the curve (AUC) was 0.99 for a validation set that explored KL divergence-based gene selection, indicating improved lung cancer prediction accuracy. Wang et al. (33) used a fully automated AI system to extract whole-lung information from CT images and predict the prognoses of patients with EGFR genotypes who had been treated with EGFR-tyrosine kinase inhibitors. The images were collected from 18,232 lung cancer patients, and the EGFR gene sequences were collected from nine cohorts in China and the United States. Kaplan–Meier analysis revealed that the AUC of fully automated AI systems ranged from 0.748 to 0.813 among the different cohorts, which was better than the AUCs of commonly used tumor-focused deep learning models. The 29 prognostic deep learning features of fully automated AI systems identified patients with EGFR mutations that exhibited a high risk of tyrosine kinase inhibitor resistance.

### 3.6 Lung cancer staging

Accurate tumor staging helps clinicians choose appropriate treatment plans. Compared with AI, imaging physicians assess tumor stages more accurately because the physicians explore both local invasion and distant metastases using images collected via multiple modalities. AI lung cancer staging (11) principally focuses on lymph node and distant metastases based on their primary tumor characteristics; it can also focus solely on lymph nodes to determine whether they are benign or malignant. Although imaging physicians are currently irreplaceable, there is evidence (34–36) that AI can provide assistance for those physicians. Yang et al. (37) evaluated 96 lung cancer patients with surgically and pathologically confirmed peripheral non-small cell lung cancer, all of whom underwent preoperative multi-detector CT and were staged using the TNM system. Two methods of maximum lesional diameter assessment (manual physician measurement and AI measurement) were used for preliminary preoperative T staging. The physician accuracy was 67.71%, whereas the AI accuracy was 83.33%. Pathological T staging consistency between the two methods was good; however, the AI CT-based T staging was more accurate, reproducible, and stable.

### 3.7 Lung cancer treatment

Watson for Oncology, an outstanding medical AI application that became available in China in 2017, rapidly and accurately develops standardized treatment plans for tumor patients; such plans are consistent with the plans established by clinical oncologists (38). Lee et al. (39) developed Seq2Seq, a deep learning algorithm that predicts weekly anatomical changes in lung tumors and the esophagus during radical radiotherapy; it incorporates potential tumor shrinkage into predictive treatment planning. The trained Seq2Seq algorithm was used to evaluate 60 patients, and its performance was very good.

### 3.8 Prediction of prognosis

AI facilitates early and accurate identification and diagnosis of individuals at high risk of lung cancer, and it also accurately predicts prognoses, aiding treatment selection. Miller et al. (40) used a new, integrated machine-learning approach to accurately predict patient survival based on metabolomics data from tumor core biopsies. Wei-Ning et al. (41) used an AI-assisted diagnostic system to extract features from lung CT images of 162 patients with adenocarcinomas who exhibited GGNs. The 5-year postoperative overall survival and relapse-free survival were better in a pure GGN group than in a mixed GGN group. Imaging microfeatures (e.g., microvascular clusters, nodule volumes, nodule lengths, and nodule diameters) were independent risk factors for poor survival. These features, as well as nodule central density and lymph node metastasis, were also independent risk factors for poor relapse-free survival, confirming that AI-assisted diagnosis could effectively predict the prognosis of GGN-type lung adenocarcinoma and support personalized treatment planning. Such study will also facilitate lung cancer prevention.

## 4 Applications of AI in other cancers

The results of the artificial intelligence software for skin cancer diagnosis developed by the research team of Stanford University in the United States were published in the journal Nature in January 2017 (42). The AI software requires the completion of three diagnostic tasks: identifying keratocytoma, identifying melanoma, and classifying melanoma using dermoscopic images. The researchers measured the performance of the algorithm through a sensitivity–specificity curve. In three diagnostic tasks, the AI performed close to that of a human dermatologist, with a sensitivity of 91%. The development of artificial intelligence software will bring revolutionary intercommunication to digital pathology research.

An AI-assisted diagnostic system developed by Dutch researchers can assess and identify the extent of lesions based on Barrett’s esophagus (43). In the research and development stage, under the guidance of endoscopy experts, the location of the lesion in the endoscopic picture is identified in combination with the histopathological diagnosis results, and then, the AI-assisted diagnosis system is used for in-depth Xi. Finally, the system compared with 53 endoscopists from 4 countries to evaluate 160 lesion countries, and the results showed that the AI-assisted diagnosis system was more accurate in predicting the lesion extent than 53 endoscopists, and all lesion ranges depicted overlapped with the range depicted by endoscopists, and the optimal biopsy site was determined in more than 92% of the cases.

## 5 Discussion

AI detection research models for lung cancer have been widely developed, and these models will be objective, efficient, multi-angle, and repeatable technical means applied to the diagnosis of lung cancer, can greatly alleviate the work pressure of clinicians, reduce doctors due to fatigue caused by misdiagnosis, may change the current medical model, is expected to make doctors work as a decision maker.

Although the effectiveness of AI in the diagnosis of lung cancer has been preliminarily verified, it is still in the stage of clinical exploration, and many aspects need to be improved: (1) The AI-assisted diagnosis system based on CT images still has missed diagnosis and misdiagnosis of benign and malignant lung cancer, which may be caused by incomplete information extraction of CT imaging data of lung cancer patients such as image features and typical manifestations and may affect the differential diagnosis results of lung cancer types by this system. (2) In a daily imaging diagnosis study, radiologists need to conduct a comprehensive evaluation and diagnosis of a lung CT image data, including determining whether there are pulmonary nodules, emphysema, pneumonia, mediastinal lymph node enlargement, and heart macrovascular lesions and then make a comprehensive evaluation. The single-task AI-assisted diagnosis system for lung cancer is obviously unable to meet the needs of comprehensive clinical study, and it is necessary to further develop a multi-task and multi-threaded real-world diagnosis system. (3) CT image data are the core resource required by AI algorithm, but at present, the degree of data sharing and interoperability among hospitals is low, and it is difficult for AI research and development companies to obtain large-scale multi-center data. Moreover, the annotation of CT images is often controversial; different countries, international organizations, societies, and hospitals may implement different standards, and understanding of signs of different doctors is not uniform. Therefore, it is imperative to establish a standard CT image database. (4) The current law does not clearly specify the scope of responsibility that AI should bear. At present, the mistakes made by AI in medical services need to be borne by doctors, but doctors cannot identify the quality of products, which makes it more difficult to assess the responsibility of AI. In the future, the responsibility of hospitals, doctors, and AI companies should be divided from the legal level, so as to urge enterprises to improve the performance of products and protect patient privacy.

Similarly, at this stage, the progress of artificial intelligence pathology diagnosis is still mostly in the laboratory research stage and has not really entered the clinic, and its limitations are as follows: (1) Data quality problems: At present, specimen processing, section staining, and image annotation have not yet formed standardized processes, and the amount of data used for artificial intelligence training is insufficient, which affects the reliability of diagnosis. (2) Data integration problem: At present, the data of artificial intelligence models mainly come from pathological sections but do not combine the symptoms, signs, and other test results of patients, which weakens the accuracy of diagnosis.

Thus far, AI is not widely used in China; however, AI is increasingly used to aid medical care, develop medicines, optimize health insurance, assist image recognition, support pathological and auxiliary diagnoses, predict prognoses, and construct disease databases. AI improves diagnostic accuracy and thus alleviates the imbalances, affecting medical and healthcare supply and demand in China. More cooperation among researchers is needed to promote rapid AI development. Although AI cannot completely replace clinical decision-making, it can serve as a valuable clinical assistant. Doctors must build appropriate emerging technologies into their clinical practices to promote the synergistic development of AI and medical care. In the near future, AI will presumably facilitate major medical breakthroughs.

## Author contributions

WL: Methodology, Resources, Writing – review & editing. NS: Methodology, Validation, Writing – original draft. LZ: Data curation, Writing – original draft. XW: Data curation, Funding acquisition, Writing – original draft. BC: Project administration, Writing – review & editing. ZL: Conceptualization, Funding acquisition, Project administration, Supervision, Writing – review & editing. CY: Conceptualization, Writing – review & editing.

## References

[1] Siegel RL Miller KD Jemal A. Cancer statistics, 2020 [J]. CA Cancer J Clin. (2020) 70:7–30. doi: 10.3322/caac.21590, PMID: 31912902

[2] Mederos N Friedlaender A Peters S Addeo A. Epidemiology, molecular genetics, and gender-specific aspects of outcome: lung cancer. esmo Open. (2020) 5:e000796. doi: 10.1136/esmoopen-2020-000796, PMID: 33148544 PMC7643520

[3] Zeng H Zheng R Guo Y Zhang S Zou X Wang N. Cancer survival in China, 2003–2005: a population‐based study. Int J Cancer. (2015) 136:1921–30. doi: 10.1002/ijc.29227 25242378

[4] Sim Y Chung MJ Kotter E Yune S Kim M do S. Deep convolutional neural network-based software improves radiologist detection of malignant lung nodules on chest radiographs [J]. Radiology. (2020) 294:199–209. doi: 10.1148/radiol.2019182465, PMID: 31714194

[5] Coudray N Ocampo PS Sakellaropoulos T Narula N Snuderl M Fenyö D. Classification and mutation prediction from non-small cell lung cancer histopathology images using deep learning [J]. Nat Med. (2018) 24:1559–67. doi: 10.1038/s41591-018-0177-5, PMID: 30224757 PMC9847512

[6] Song L Zhu ZC Jiang L Zhao L Yang QL Sui X. Preliminary investigation on the value of CT imaging histopathology in predicting fusion gene expression in lung adenocarcinoma [J] (in Chinese). Chin J Radiol. (2019) 53:963–7. doi: 10.3760/cma.j.issn.1005-1201.2019.11.007

[7] He L Huang YQ Ma ZEL Liang CS Huang XM Cheng ZX. The value of CT imaging histology in clinical staging of non-small cell lung cancer [J] (in Chinese). Chin J Radiol. (2017) 51:906–11. doi: 10.3760/cma.j.issn.1005-1201.2017.12.004

[8] Banna GL Olivier T Rundo F Malapelle U Fraggetta F Libra M. The promise of digital biopsy for the prediction of tumor molecular features and clinical outcomes associated with immunotherapy [J]. Front Med. (2019) 6:172. doi: 10.3389/fmed.2019.00172, PMID: 31417906 PMC6685050

[9] Wang S Wang S Liu Z Zhang Q. A role distinguishing Bert model for medical dialogue system in sustainable smart city [J]. Sustain Energy Technol Assess. (2023) 55:102896. doi: 10.1016/j.seta.2022.102896

[10] Gao C Wang SW Xu MS. Artificial intelligence study of lung cancer images [J] (in Chinese). Chin J Integr Chin West Med Imag. (2020) 18:219–23. doi: 10.3969/j.issn.1672-0512.2020.03.001

[11] Li Q Liu Y Zhang YW Ye ZX. Research progress of artificial intelligence in lung tumor imaging diagnosis [J] (in Chinese). Chin Cancer Clin. (2020) 47:55–9. doi: 10.3969/j.issn.1000-8179.2020.02.987

[12] Yan W Li NN Zhang YZ Maode XY. Pathology histology in the era of artificial intelligence [J] (in Chinese). J Clin Exp Pathol. (2018) 34:661–3. doi: 10.13315/j.cnki.cjcep.2018.06.017

[13] Gurcan MN Boucheron LE Can A Madabhushi A Rajpoot NM Yener B. Histopathological image analysis: a review [J]. IEEE Rev Biomed Eng. (2009) 2:147–71. doi: 10.1109/RBME.2009.2034865, PMID: 20671804 PMC2910932

[14] Eraslan G Avsec Z Gagneur J Theis FJ. Deep learning: new computational modelling techniques for genomics [J]. Nat Rev Genet. (2019) 20:389–403. doi: 10.1038/s41576-019-0122-6 30971806

[15] Scholten ET Horeweg N de Koning HJ Vliegenthart R Oudkerk M Mali WPTM. Computed tomographic characteristics of interval and post screen carcinomas in lung cancer screening. Eur Radiol. (2015) 25:81–8. doi: 10.1007/s00330-014-3394-4 25187382

[16] Zhigai Z Xuanqi S Liandi L. Analysis of the value of Ai recognition technology in early lung cancer diagnosis [J] (in Chinese). Chin Pract Med. (2020) 15:58–60. doi: 10.14163/j.cnki.11-5547/r.2020.22.023

[17] Mahmood SA Ahmed HA. An improved CNN-based architecture for automatic classification of lung nodules [J]. Med Biol Eng Comput. (2020) 60:1977–86. doi: 10.1007/s11517-022-02578-0 35524089

[18] Rabbani M Kanevsky J Kafi K Chandelier F Giles FJ. Role of artificial intelligence in the care of patients with non-small cell lung cancer [J]. Eur J Clin Investig. (2018) 48:e12901. doi: 10.1111/eci.12901, PMID: 29405289

[19] Yang Y Li XQ Han ZB Fu JP Gao B. A study of primary and malignant lung nodules classification based on three-dimensional multi-view squeeze and excitation convolutional neural network [J]. Seabrook Int J. (2022) 39:452–61. doi: 10.7507/1001-5515.202110059 PMC10950768 35788514

[20] Ma H Guo H Zhao M Qi S Li H Tian Y. Automatic pulmonary ground-glass opacity nodules detection and classification based on 3D neural network [J]. Med Phys. (2022) 49:2555–69. doi: 10.1002/mp.15501 35092608

[21] Liu M Xianglin Z. Study on the value of artificial intelligence for early lung cancer screening [J] (in Chinese). Health Vision. (2021) 10:004.

[22] Wu JC Li T Li XD Zhuo Y Zhang YJ Liu JY. Study on the factors influencing the prediction of lung nodule growth based on artificial intelligence follow-up [J] (in Chinese). Chin Gen Med. (2022) 25:2115–20. doi: 10.12114/j.issn.1007-9572.2022.0005

[23] Yu H Zhang Q Yang LT. An edge-cloud-aided private high-order fuzzy C-means clustering algorithm in smart healthcare. IEEE/ACM Trans Comput Biol Bioinform. (2023) PP:1–10. doi: 10.1109/TCBB.2022.3233380, PMID: 37018339

[24] Guo Y Li RB Liu SH Wei LJ Cui FS. Research on lung cancer image assisted diagnosis application based on machine learning [J] (in Chinese). China Med Equipment. (2021) 18:124–8. doi: 10.3969/J.ISSN.1672-8270.2021.03.030

[25] Mei N. Application of artificial intelligence technology in medical imaging [J]. Fam Med (in Chinese). (2019) 2:78. doi: CNKI:SUN:YYJT.0.2019-02-087

[26] Feng J Jiang J. Deep learning-based chest CT image features in diagnosis of lung cancer [J]. Comput Math Methods Med. (2022) 2022:1–7. doi: 10.1155/2022/4153211 PMC8791752 35096129

[27] Shi F Chen B Cao Q Wei Y Zhou Q Zhang R. Semi-supervised deep transfer learning for benign-malignant diagnosis of pulmonary nodules in chest CT images [J]. IEE Trans Med Imaging. (2022) 41:771–81. doi: 10.1109/TMI.2021.3123572, PMID: 34705640

[28] Fang W Zhang G Yu Y Chen H Liu H. Identification of pathological subtypes of early lung adenocarcinoma based on artificial intelligence parameters and Ct signatures [J]. Biosci Rep. (2022) 42:1–28. doi: 10.1042/BSR20212416 PMC8766821 35005775

[29] Yu H Yang LT Zhang Q Armstrong D Deen MJ. Convolutional neural networks for medical image analysis: state-of-the-art, comparisons, improvement and perspectives. Neurocomputing. (2021) 444:92–110. doi: 10.1016/j.neucom.2020.04.157

[30] Wang J Yang Z Zhu Q Jia YH Chen L Liang ZX. A preliminary application of an artificial intelligence-based cytopathological diagnosis system in lung cancer diagnosis [J] (in Chinese). J PLA Med Coll. (2020) 41:897–900. doi: 10.3969/j.issn.2095-5227.2020.09.012

[31] Chen YY Kong WZ Wu HQ Ji JL. A deep learning-based artificial intelligence-assisted diagnosis of lung cancer pleural fluid exfoliation cytology [J] (in Chinese). Chin Clin Med. (2022) 29:396–400. doi: 10.12025/j.issn.1008-6358.2022.20220658

[32] Liu S Yao W. Prediction of lung cancer using gene expression and deep learning with kl divergence gene selection [J]. BMC Bioinform. (2022) 23:175. doi: 10.1186/s12859-022-04689-9, PMID: 35549644 PMC9103042

[33] Wang S Yu H Gan Y Wu Z Li E Li X. Mine whole-lung information by artificial intelligence for predicting EGFR genotype and targeted treatment response in lung cancer: a multicohort study [J]. Lancet Digit Health. (2022) 4:e309–19. doi: 10.1016/S2589-7500(22)00024-3 35341713

[34] Ferreira-Junior JR Koenigkam-Santos M Magalhães Tenório AP Faleiros MC Garcia Cipriano FE Fabro AT. CT-based radiomics for prediction of histologic subtype and metastatic disease in primary malignant lung neoplasms. Int J Comput Assist Radiol Surg. (2020) 15:163–72. doi: 10.1007/s11548-019-02093-y, PMID: 31722085

[35] Bayanati H Thornhill RE Souza CA Sethi-Virmani V Gupta A Maziak D. Quantitative CT texture and shape analysis: can it differentiate benign and malignant mediastinal lymph nodes in patients with primary lung cancer? Eur Radiol. (2015) 25:480–7. doi: 10.1007/s00330-014-3420-6, PMID: 25216770

[36] Moitra D Mandal RK. Automated AJCC (7th edition) staging of non-small cell lung cancer (NSCLC) using deep convolutional neural networks (CNN) and recurrent neural networks (RNN) [J]. Health Inf Sci Syst. (2019) 7:14. doi: 10.1007/s13755-019-0077-1 31406570 PMC6667593

[37] Yang LL Wang F Wang ZR Yang YB Ha RS Zhang M. A controlled study of imaging T-staging and pathological T-staging of non-small cell lung cancer based on artificial intelligence [J]. Ningxia Med J (in Chinese). (2021) 43:782–4. doi: 10.13621/j.1001-5949.2021.09.0782

[38] Chen-Xing H Xiao-Chun Z. A study on the consistency of artificial intelligence and oncologists' choice of lung cancer treatment plan [D] (in Chinese). Qingdao: Qingdao University (2020).

[39] Lee D Hu YC Kuo L Alam S Yorke E Li A. Deep learning driven predictive treatment planning for adaptive radiotherapy of lung cancer [J]. Radiother Oncol. (2022) 169:57–63. doi: 10.1016/j.radonc.2022.02.013, PMID: 35189155 PMC9018570

[40] Miller HA van Berkel VH Frieboes HB. Pooled machine learning analysis of tumor core biopsy metabolomic data for lung cancer survival prediction and biomarker identification [J]. Metabolomics. (2022) 18:57. doi: 10.1007/s11306-022-01918-3 35857204 PMC9737952

[41] Wei N Lin RJ Ma MJ Chen C Han B. Relationship between imaging microfeatures of artificial intelligence-aided diagnostic system and prognosis of ground glass nodule-like lung cancer [J] (in Chinese). Cancer Control Res. (2021) 48:877–82. doi: 10.3971/j.issn.1000-8578.2021.21.0255

[42] Esteva A Kuprel B Novoa RA Ko J Swetter SM Blau HM. Dermatologist-level classification of skin cancer with deep neural networks. Nature. (2017) 542:115–8. doi: 10.1038/nature21056, PMID: 28117445 PMC8382232

[43] De Groof AJ Struyvenberg MR van der Putten J van der Sommen F Fockens KN Curvers WL. Deep-learning system detects neoplasia in patients with Barrett's esophagus with higher accuracy than endoscopists in a multi-step training and validation study with benchmarking [J]. Gastroenterology. (2020) 158:915–929.e4. doi: 10.1053/j.gastro.2019.11.030 31759929

